# Potential application of bisoprolol derivative compounds as antihypertensive drugs: synthesis and *in silico* study

**DOI:** 10.1098/rsos.231112

**Published:** 2023-12-20

**Authors:** Ni Putu Sani Oktaviani, Atthar Luqman Ivansyah, Muhammad Yogi Saputra, Nurrahmi Handayani, Nurdiani Fadylla, Deana Wahyuningrum

**Affiliations:** ^1^ Department of Chemistry, Organic Chemistry Division, Faculty of Mathematics and Natural Sciences, Institut Teknologi Bandung, Bandung 40132, Indonesia; ^2^ Master Program in Computational Science, Faculty of Mathematics and Natural Sciences, Institut Teknologi Bandung, Bandung 40132, Indonesia; ^3^ Instrumentation and Computational Physics Research Group, Department of Physics, Faculty of Mathematics and Natural Sciences, Institut Teknologi Bandung, Bandung 40132, Indonesia; ^4^ Analytical Chemistry Division, Department of Chemistry, Institut Teknologi Bandung, Bandung 40132, Indonesia; ^5^ Department of Chemistry, Faculty of Sciences, Institut Teknologi Sumatera (ITERA), Jalan Terusan Ryacudu, Way Hui, Kecamatan Jati Agung, Lampung Selatan 35365, Indonesia; ^6^ PT. Kimia Farma Tbk, Jl Raya Banjaran KM 16 16 Banjaran, Kab Bandung, Jawa Barat, Indonesia

**Keywords:** bisoprolol, derivative, antihypertensives, molecular docking, organic synthesis

## Abstract

Two bisoprolol derivatives, *N*-acetyl bisoprolol and *N*-formyl bisoprolol, belonging to the beta-blocker class of antihypertensive drugs, were synthesized using acetylation and formylation reactions. The yields of the reactions were determined to be 32.40% for *N*-acetyl bisoprolol and 20.20% for *N*-formyl bisoprolol. *In silico* methods such as molecular docking, molecular dynamics simulation and SwissADME prediction were employed to evaluate the potential of these bisoprolol derivatives as antihypertensive drugs. These methods were used to assess the interaction between the bisoprolol derivatives and various receptors associated with hypertension, including human angiotensin I-converting enzyme (PDB ID: 1O8A), renin (PDB ID: 2V0Z), beta-1 adrenergic receptors (PDB ID: 4BVN, 7BVQ), voltage-dependent L-type calcium channel subunit alpha-1S (PDB ID: 6JP5) and mineralocorticoid receptor (PDB ID: 6L88). Our results demonstrated the highest binding energy when bisoprolol and its derivatives bound to 4BVN, with binding energy values of 6.74 kcal mol^−1^, 7.03 kcal mol^−1^ and 7.63 kcal mol^−1^ for bisoprolol, *N*-acetyl bisoprolol and *N*-formyl bisoprolol, respectively. The stability of these complexes was confirmed by molecular dynamics simulations, with a root-mean-square deviation value of approximately 2. Furthermore, the SwissADME results indicated that both derivatives exhibited similar properties to the reference drug bisoprolol.

## 1. Introduction

High blood pressure is a significant risk factor for cardiovascular disease and a leading cause of mortality. Annually, hypertension contributes to approximately 9.4 million deaths worldwide [1]. Furthermore, it is a major modifiable risk factor for heart disease, stroke, end-stage renal failure and peripheral vascular disease [2]. According to the World Health Organization, approximately 1.28 billion individuals between the ages of 30 and 79 suffer from hypertension. Consequently, reducing the prevalence of hypertension by 33% between 2010 and 2030 has become one of the global targets for non-communicable diseases [3]. By effectively lowering blood pressure, it is possible to decrease cardiovascular morbidity, mortality and overall mortality rates. While lifestyle modifications are essential for managing hypertension, the majority of hypertensive individuals require antihypertensive medications to effectively lower and maintain their blood pressure within an acceptable range.

Various commercially available antihypertensive drugs, including beta-blockers, angiotensin I-converting enzyme (ACE) inhibitors and calcium channel blockers, have been used to mitigate hypertension by inhibiting key enzymes and proteins involved in blood pressure regulation [4]. Among these drugs, bisoprolol stands out as an effective treatment for hypertension [5]. It has demonstrated the ability to significantly reduce long-term mortality from heart disease and myocardial infarction in high-risk patients following cardiac vascular surgery [6]. Bisoprolol is classified as a highly cardioselective beta-1 blocker, making it a potent antihypertensive agent compared with other cardioselective beta-blockers such as atenolol, metoprolol, betaxolol, esmolol, acebutolol and nebivolol. However, a notable drawback of bisoprolol, being a beta-1 blocker, is its potential to reduce myocardial cell oxygen consumption [7]. Consequently, there arises a need to modify the drug consumption of bisoprolol.

To enhance the effectiveness of bisoprolol in treating hypertension, modifications have been implemented by combining it with other antihypertensive drugs. One such combination involves pairing bisoprolol with hydrochlorothiazide, a diuretic known to lower blood pressure by approximately 40% [8]. Additionally, bisoprolol can be combined with amlodipine, a calcium channel blocker that has been shown to reduce blood pressure by approximately 39% [8,9]. The rationale behind combining these medications is that using two drugs in low doses can enhance the tolerability of side effects compared with increasing the dosage of a single drug [10,11]. By employing combination therapy, the aim is to optimize the antihypertensive effects while minimizing adverse reactions associated with higher doses of individual drugs.

In addition to combination therapies, the development of antihypertensive drugs can also involve the synthesis of derivative compounds. For instance, Masaret *et al*. [12] successfully developed antihypertensive drug compounds by synthesizing pyridone derivatives. Furthermore, there have been advancements in the development of antihypertensive drug compounds through synthesis and molecular docking methods. Studies have explored the potential of xanthone derivatives as antihypertensive drugs using this approach [13]. Similarly, the synthesis and molecular docking of pyrimidine derivatives have shown promise in their potential as antihypertensive agents [14,15]. These efforts highlight the ongoing exploration of derivative compounds as a means to develop novel and effective antihypertensive drugs.

In this study, we aimed to synthesize bisoprolol derivatives, namely *N*-acetyl bisoprolol and *N*-formyl bisoprolol, through acetylation and formylation reactions to explore their potential as antihypertensive drugs. To the best of our knowledge, no previous research has investigated the antihypertensive properties of these specific bisoprolol derivatives. Hence, we conducted molecular docking, molecular dynamics (MD) simulations and SwissADME prediction to screen and evaluate the efficacy of these synthesized compounds. These computational methods were employed to assess the interaction between the bisoprolol derivatives and various receptors related to hypertension. Our study represents a novel approach to the evaluation of the antihypertensive potential of bisoprolol derivatives and provides valuable insights into their potential applications in the treatment of hypertension.

## 2. Material and methods

### 2.1. Materials

Bisoprolol base (98.5%) was acquired from Mikromol, while other reagents such as acetic anhydride (98.5%), formic acid (98–100%) and ZnCl_2_ (98–100.5%) were obtained from Merck. All common solvents used in the experiments were of chemically pure and analytical research (AR) grade, sourced from Merck and Fluka and used without further purification. The ^1^H-NMR signals were measured using an Agilent NMR instrument with a frequency of 500 MHz, while the ^13^C-NMR signals were measured using an Agilent NMR instrument with a frequency of 125 MHz. Fourier transform infrared (FTIR) spectroscopy was performed using a Prestige 21 Shimadzu instrument to obtain absorption spectra of the functional groups present in the products. Pure compounds were obtained using a Shimadzu high-performance liquid chromatography (HPLC) system equipped with a photodiode array (PDA) detector.

### 2.2. Synthesis of bisoprolol derivatives

#### 2.2.1. Synthesis of *N*-acetyl bisoprolol

To begin the synthesis, 1 g (3.07 mmol) of bisoprolol was dissolved in 2 ml of dichloromethane. Subsequently, 0.575 ml (6.08 mmol) of acetic anhydride was added to the solution. The reaction mixture was allowed to react for 30 min. After completion of the reaction, the mixture was dissolved in dichloromethane and washed with saturated sodium bicarbonate solution until reaching a pH of 7–8. The organic phase was then washed with two portions of 10 ml of distilled water and dried using sodium sulfate to remove any remaining water. The resulting reaction mixture was concentrated, and to confirm the formation of the product, a thin layer chromatography (TLC) test was performed. For further purification, the product was subjected to preparative TLC using an appropriate solvent system.

The obtained product was subsequently identified and characterized using various techniques. TLC was used to confirm the presence of the product, while FTIR spectroscopy was employed to analyse the functional groups present. ^1^H-NMR and ^13^C-NMR spectroscopies were used to provide further structural information about the product.

#### 2.2.2. Synthesis of *N*-formyl bisoprolol

A mixture containing 0.04 g of ZnCl_2_ (10 mol%) and 0.288 ml (7.63 mmol) of formic acid was added to 1 g (3.07 mmol) of bisoprolol (with a molar ratio of 1 mol bisoprolol to 2.48 mol formic acid). The reaction mixture was heated using the reflux method at a temperature of 70–75°C. Throughout the reaction, TLC was periodically performed to monitor the progress of the reaction. Initially, the reaction mixture appeared colourless and slightly cloudy. After 2 h of heating, the mixture turned dark yellow.

Following the completion of the reaction, the reaction mixture was dissolved in dichloromethane and washed with saturated sodium bicarbonate solution until reaching a pH of 7–8. A precipitate of ZnCl_2_ formed between the aqueous and organic phases. The ZnCl_2_ precipitate was separated through vacuum filtration. The organic phase was then washed with two portions of 10 ml distilled water and concentrated. The resulting product was characterized using TLC, FTIR, ^1^H-NMR and ^13^C-NMR to confirm its identity and determine its structural properties.

### 2.3. *In silico* studies

#### 2.3.1. Molecular docking

##### 2.3.1.1. Protein preparation

The three-dimensional structures of the human ACE protein (PDB ID: 1O8A), renin (PDB ID: 2V0Z), beta-1 adrenergic receptor (PDB ID: 4BVN, 7BVQ), voltage-dependent L-type calcium channel alpha-1S subunit (PDB ID: 6JP5) and mineralocorticoid receptor (PDB ID: 6L88) were obtained from the Protein Data Bank (www.rcsb.org). To prepare these protein structures for molecular docking simulations, YASARA software v. 21.12.19 [16] was used.

Protein preparation involved several steps using the YASARA software. Firstly, water molecules were removed from the protein structures. Then, unwanted atoms and molecules were cleaned from the proteins. Subsequently, energy minimization was performed to optimize the protein structures and obtain stable conformations. This step was accomplished using the em_run.mcr macro in the YASARA software.

By applying these protein preparation steps, the structures of the proteins were optimized and made suitable for subsequent molecular docking simulations. This ensured that the proteins were in an appropriate conformation for interacting with the bisoprolol derivatives and assessing their binding interactions.

##### 2.3.1.2. Ligand preparation

Ligand structures were prepared using MarvinSketch software (v. 20.14). The ligands were the synthesized compounds, i.e. *N*-acetyl bisoprolol and *N*-formyl bisoprolol, which were identified for their activity as antihypertensive drugs. After each ligand structure was made, the energy of each ligand was minimized to obtain a stable structure using the em_run.mcr macro on YASARA.

##### 2.3.1.3. Docking ligand to the receptor

Molecular docking simulations were carried out using the dock_run.mcr macro with the AMBER03 forcefield. The chosen docking method was blind docking, which involves docking the ligand to the entire surface of the protein without prior knowledge of the active site. This approach allows for the identification of the optimal binding site for the ligand on the target receptor. Blind docking offers the advantage of exploring potential binding regions beyond known active sites. During the molecular docking simulations, the dock_run.mcr macro generated data in the form of binding energy, also referred to as the docking score or interaction energy, between the ligand and receptor molecules. A more positive binding energy and a smaller dissociation constant indicate a stronger binding affinity between the ligand and receptor [17].

To analyse and visualize the interaction between the ligand and protein, Ligplus+ and Origin software were employed. These tools allow for the examination of the binding mode and interactions, providing valuable insights into the molecular interactions and potential mechanisms of ligand–receptor binding.

#### 2.3.2. Molecular dynamics

MD simulations were performed using YASARA software version 21.12.19 with the AMBER14 forcefield. The md_run.mcr macros were employed to carry out these simulations. The integration time, or timestep, used was 2 × 1.25 fs, and the simulation duration was set to 100 ns. During the simulations, data and trajectories were recorded at regular intervals of 100 ps. This allowed for the monitoring and analysis of the system's behaviour throughout the simulation.

To evaluate the stability and dynamics of the system, parameters such as the root-mean-square deviation (RMSD) value and the number of hydrogen bonds were calculated. The RMSD value provides information about the structural changes and fluctuations of the system throughout the simulation. The number of hydrogen bonds can give insights into the strength and stability of the protein–ligand interactions. The data obtained from the MD simulation, including the RMSD values and the number of hydrogen bonds, were analysed and visualized using Origin software. This allowed for a comprehensive understanding of the system's behaviour and the dynamics of the ligand–receptor complex during the simulation.

#### 2.3.3. Lipinski's rule and prediction of absorption, distribution, metabolism, excretion, and toxicity

To assess the absorption, distribution, metabolism, excretion, and toxicity (ADMET) properties of *N*-acetyl bisoprolol and *N*-formyl bisoprolol compounds, the SwissADME Web server was used. The Web server employs various computational algorithms to predict important drug-like properties. The structures of both *N*-acetyl bisoprolol and *N*-formyl bisoprolol compounds were submitted to the SwissADME Web server. The server automatically converted the structures into Simplified Molecular Input Line Entry System (SMILES) notation, which is a standard representation of molecular structures. Using the SwissADME Web server, several ADMET properties were calculated, including Lipinski's rule of 5, which assesses drug-likeness based on parameters related to molecular weight, lipophilicity, and hydrogen bonding capability [18]. Additionally, gastrointestinal (GI) absorption, blood–brain barrier (BBB) permeability and cytochrome inhibition were predicted [19,20]. These predictions provide valuable insights into the pharmacokinetic and safety profiles of the *N*-acetyl bisoprolol and *N*-formyl bisoprolol compounds.

## 3. Results and discussion

### 3.1. Synthesis of bisoprolol derivatives

The synthesis of bisoprolol derivates has been carried out to produce compounds *N*-acetyl bisoprolol (**3**) and *N*-formyl bisoprolol (**5**) from the same precursor, bisoprolol (**1**). Compound **3** has been synthesized using acetylating agent acetic anhydride (**2**). The formation of compound **3** was confirmed by its FTIR spectrum (electronic supplementary material, figure S1) with C=O characteristic peak at a wavenumber of 1612 cm^−1^, by ^1^H-NMR and ^13^C-NMR (electronic supplementary material, figures S2 and S3) spectra which confirmed the presence of an acetyl group, indicated by a methyl proton signal with singlet multiplicity at 2.21 ppm and a C=O carbon signal at a chemical shift 173.62 ppm.

Compound **3** has 97.88% purification which was analysed by the HPLC instrument with the chromatogram shown in electronic supplementary material, figure S4. On the other hand, compound **5** was synthesized by formic acid (**4**). The formation of compound **5** was confirmed through the FTIR spectrum which displayed a peak corresponding to the C=O group at a wavenumber of 1660 cm^−1^ (electronic supplementary material, figure S5). Furthermore, the presence of a formyl group was verified by observing a proton aldehyde signal at 8.26 ppm and a carbon signal corresponding to C=O at 164.87 ppm in the ^1^H-NMR and ^13^C-NMR spectra, respectively (electronic supplementary material, figures S6 and S7). Synthesized compound **5** has 96.32% purification which was analysed by the HPLC instrument (electronic supplementary material, figure S8).

The yields for the two compounds were 32.40% and 20.2% for compounds **3** and **5**, respectively. The yield of the reaction can be influenced by the selectivity of the reaction, leading to the formation of side products that may be considered impurities. In the synthesis reaction of compound **3**, the reaction is not selective due to the presence of a hydroxy functional group in bisoprolol that is also reactive. To address this issue, the addition of a MgO catalyst in THF:H_2_O (4 : 1) medium can be employed to promote a selective reaction specifically targeting the secondary amine groups [21]. On the other hand, during the synthesis of compound **5**, the use of ethyl methanoate or methyl methanoate can prevent the protonation of the secondary amine group by formic acid, rendering the group unreactive [22].

### 3.2. Molecular docking

Molecular docking simulations were conducted to assess the binding potential of three ligands, namely compounds **1**, **3** and **5**, to six target proteins with the PDB codes 1O8A, 2V0Z, 4BVN, 6JP5, 6L88 and 7BVQ. Although bisoprolol is a beta-1 selective inhibitor, its potency to inhibit another receptor was evaluated to see whether it has better activity with several receptors through simulation and the results determined that bisoprolol not only has potency as a beta-1 selective inhibitor but also can have potency to inhibit another similar receptor. Bisoprolol has adverse inotropic and chronotropic effects. In its actions, bisoprolol reduces the oxygen consumption of myocardial cells, relieving the heart's workload. Bisoprolol as a B1 inhibitor is also present in the juxtaglomerular cells of the human kidneys. By blocking these receptors, bisoprolol reduces the release of renin, thereby blocking the activation of the renin–angiotensin system [23].

**Figure 1.**
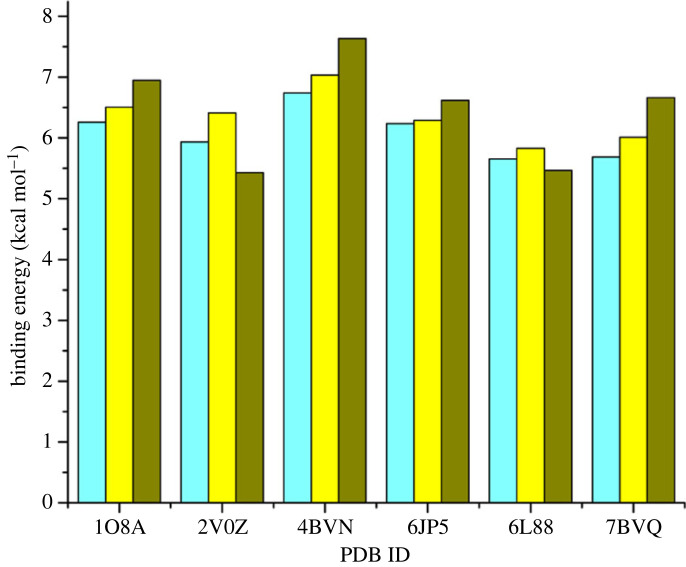
Binding energy of bisoprolol (compound **1**, blue), *N*-acetyl bisoprolol (compound **3**, yellow) and *N*-formyl bisoprolol (compound **5**, green) with six target proteins through molecular docking simulations.

The simulations yielded binding energy values, also known as docking scores, which indicate the strength of interaction between the ligands and the target receptors. In YASARA software, a more positive binding energy indicates a stronger interaction between ligand and receptor. The interactions between the ligands and the receptors were evaluated based on hydrogen bonds and hydrophobic interactions. Figure 1 and table 1 present the results, displaying the binding energy values of each compound with the six target proteins. These findings provide valuable insights into the potential interactions and affinities of the ligands towards the respective receptors.

**Table 1.** Binding energy scores from molecular docking simulations.

protein	binding energy (kcal mol^−1^)		
	bisoprolol	*N*-acetyl bisoprolol	*N*-formyl bisoprolol
1O8A	6.26	6.50	6.95
2V0Z	5.93	6.41	5.43
4BVN	6.74	7.03	7.63
6JP5	6.24	6.29	6.62
6L88	5.65	5.83	5.47
7BVQ	5.69	6.01	6.66

The binding energy values of compounds **1**, **3** and **5** with proteins 1O8A, 4BVN, 6JP5 and 7BVQ showed an almost consistent trend from highest to lowest as **5** > **3** > **1**. This indicates that the two synthesized derivative compounds (**3** and **5**) have stronger binding interactions than the commercial drug bisoprolol (compound **1**). The order of binding energies is likely influenced by the involvement of a larger number of amino acids in hydrophobic interactions and the presence of hydrogen bonds in each ligand–receptor complex.

Detailed information on the hydrophobic interactions and hydrogen bonding in the ligand–receptor complexes with proteins 1O8A, 4BVN, 6JP5 and 7BVQ can be found in tables 2–5, respectively. These tables provide insights into the specific interactions that contribute to the binding strengths of the ligands with their respective target proteins.

**Table 2.** Interactions of compounds **1**, **3** and **5** with the human ACE receptor (1O8A protein).

ligand	hydrogen bond		hydrophobic interactions
	amino acid	distances	
**1**	OG1 Thr372 with O1 **1**; OG1 Thr302 with O4 **1**	3.31; 3.02	Gln369, Cys370, Val380, Asp377, Asn374, Thr171, Asn167, Asn285, Glu376, Thr166, Pro163, Glu162
**3**	NZ Lys454 with O3 **3**	3.05	Asp453, Val379, His383, Phe572, Val380, Glu376, Glu162, Asp377, Ala354, His353, Gln281, Tyr523, Phe457
**5**	—	—	Glu225, Phe570, Ser222, Tyr213, Glu411, His387, Phe391, Pro407, His410, Arg522, Asn406, Glu403, Gly404, Met223, Lys118

**Table 3.** Interactions of compounds **1**, **3** and **5** with the beta-adrenergic receptor (4BVN protein).

ligand	hydrogen bond		hydrophobic interactions
	amino acid	distances	
**1**	O Ala227 with N **1**	3.25	Ala136, Met96, Gly293, Val230, Lys290, Lys287, Met283, His286, Ile238, Ala234, Leu289, Tyr231, Leu292
**3**	ND1 His286 with O1 **3**	2.95	Lys235, Ile238, Met283, Lys287, Ala234, Lys290, Leu289, Val230, Tyr231, Ala136, Ley292, Gly293, Met296, Ala227
**5**	N Phe201 with O2 **5**; ND3 Asn310 with O3 **5**	3.21; 2.92	Asp200, Leu101, Asn329, Phe325, Val326, Cys199, Trp117, Phe306, Ser215, Ser211, Phe307, Val122

**Table 4.** Interactions of compounds **1**, **3** and **5** with the calcium channel receptor (6JP5 protein).

ligand	hydrogen bond		hydrophobic interactions
	amino acid	distances	
**1**	ND2 Asn649 with O1 **1**; ND2 Asn649 with O2 **1**	2.92; 3.17	Glu292, Thr290, Thr612, Phe645, Leu611, Gly613, Phe1054, Leu657, Leu330, Asn1058, Val1061
**3**	—	—	His198, Pro195, Gly1252, His1397, Asp1400, Pro1396, Thr1255, Glu1251, Trp1391, Tyr1386, Val332, Leu1256
**5**	—	—	Leu1256, Val1332, Pro195, Trp1391, His1397, Pro1396, Arg1389, Thr1259, Trp1258, Asp1400, Thr1255, Tyr1386, Leu1387

**Table 5.** Interactions of compounds **1**, **3** and **5** with the beta-adrenergic receptor (7BVQ protein).

ligand	hydrogen bond		hydrophobic interactions
	amino acid	distances	
**1**	NH1 Arg1038 with O1 **1**; NH1 Arg1038 with N **1**	3.14; 3.30	Arg1123, Trp1121, Gly1122, Asn1034, Val1120, Ala1050, Thr1041, Lys1037, Ala1053, Trp1057, Ser1054
**3**	N Phe1218 with O3 **3**	3.02	Trp1134, Lys1347, Asp1356, Phe1359, Val1360, Val1119, Ile1118, Asp1217, Asn1363, Cys1216
**5**	NH2 Arg1156 with O2 **5**; OG1 Thr1093 with O3 **5**	2.91; 3.09	Ala1251, Val1255, Glu1319, Ile1160, Gln1320, Leu1323, Ile1329, Ile1097, Tyr1377, Ser1380, Phe1383, Thr1325, Asn1094, Leu1326, Ala1322

The binding energies between compounds **1**, **3** and **5** with the 1O8A and 2V0Z proteins were observed in the order of **3** > **1** > **5**. Details regarding hydrophobic interactions and hydrogen bonding in the ligand–receptor complexes can be found in tables 2 and 6 for 1O8A and 2V0Z, respectively. The 1O8A and 2V0Z proteins are associated with the abnormal activity of the RAS (renin–angiotensin system) that produces angiotensin 2 [24]. Among the three simulated ligands, all demonstrated the ability to inhibit the proteins with positive binding energy values. However, the synthesized ligands (compounds **3** and **5**) exhibited better binding potential than the commercially available drug (compound **1**). Notably, compound **3** displayed the best binding potential in inhibiting both proteins, potentially countering the formation of angiotensin 2.

**Table 6.** Interactions of compounds **1**, **3** and **5** with the renin receptor (2V0Z protein).

ligand	hydrogen bond		hydrophobic interactions
	amino acid	distances	
**1**	OG1 Thr77 with O1 **1**	3.08	Asp215, Gly217, Ala218, Val120, Val30, Asp32, Tyr75, Phe117, Leu114, Gln13, Pro111
**3**	—	—	Thr77, Met289, Ser219, Gln13, Phe117, Pro111, Val30, Asp32, Tyr220, Val120, Tyr75, Gly217
**5**	N Leu324 with O2 **5**	3.21	Ala323, Asn178, His180, Tyr267, Thr268, Lys265, Glu266, Phe309, Gly177, Glu176

The 4BVN protein is a beta-adrenergic receptor responsible for increasing blood pressure when the amount of adrenaline exceeds normal limits. The three ligands simulated showed potential for inhibiting the 4BVN protein but based on the results of binding energy and non-covalent interactions, ligand **5** demonstrated the most optimal binding ability to the receptor. On the other hand, the 6JP5 protein is a calcium channel receptor. The presence of calcium entering the cells of the heart and arteries causes them to contract more forcefully. All three simulated ligands showed potential for inhibiting the 6JP5 protein, but ligand **5** displayed the most optimal binding ability.

The 6L88 protein is a mineralocorticoid receptor that is crucial in regulating blood pressure, electrolytes and fluid homeostasis. Blocking these receptors is essential in preventing cardiovascular events. Among the three simulated ligands, ligand **3** exhibited the most optimal binding ability to inhibit the 6L88 protein (table 7). As for the 7BVQ protein, it is a beta-adrenergic receptor. All three simulated ligands showed potential for inhibiting the 7BVQ protein, but ligand **5** demonstrated the most optimal binding ability.

**Table 7.** Interactions of compounds **1**, **3** and **5** with the mineralocorticoid receptor (6L88 protein).

ligand	hydrogen bond		hydrophobic interactions
	amino acid	distances	
**1**	**—**	**—**	Met777, Met959, Gly774, Trp806, Ala773, Phe829, Ser810, Leu814, Arg817, Met807, Leu769, Asn770, Cys942
**3**	**—**	**—**	Tyr944, Gln803, Cys94, Tyr804, Leu939, Phe943, Gly774, Pro957, Ala773, Met777, Met959, Ala958
**5**	**—**	**—**	Met777, Ala773, Phe829, Leu769, Asn770, Pro957, Tyr944, Leu960, Met959, Gly774

The binding energy, or docking score, indicates the strength of a ligand's interaction with a receptor's binding pocket. In various computational studies exploring potential antihypertensive drugs through molecular docking, the obtained docking scores have shown variations. For example, pyridone derivatives inhibiting calcium channel receptors had docking scores ranging from −5.26 to −7.16 kcal mol^−1^ [12], xanthone-derived compounds exhibited scores between −8.6 and −9.1 kcal mol^−1^ [13], and pepsin ligands from whey protein hydrolysate from camel exhibited scores from −9.22 to −12.36 kcal mol^−1^ targeting the human ACE receptor [4]. Negative docking scores represent binding energy, with more negative values indicating stronger interactions with the receptor's binding pocket.

However, in this research, the obtained binding energy values ranged from 5.653 to 7.633 kcal mol^−1^. Here, positive values indicate stronger bond strength between the ligands and the receptor's binding pocket. Despite the positive values, the binding energy range aligns with previous studies investigating the potential of compounds as antihypertensive drugs based on binding energy values [4,12,13]. These studies used different ligands and receptors, but their results demonstrated similar value ranges for binding energy, indicating their activity as potential antihypertensive agents.

**Figure 2.**
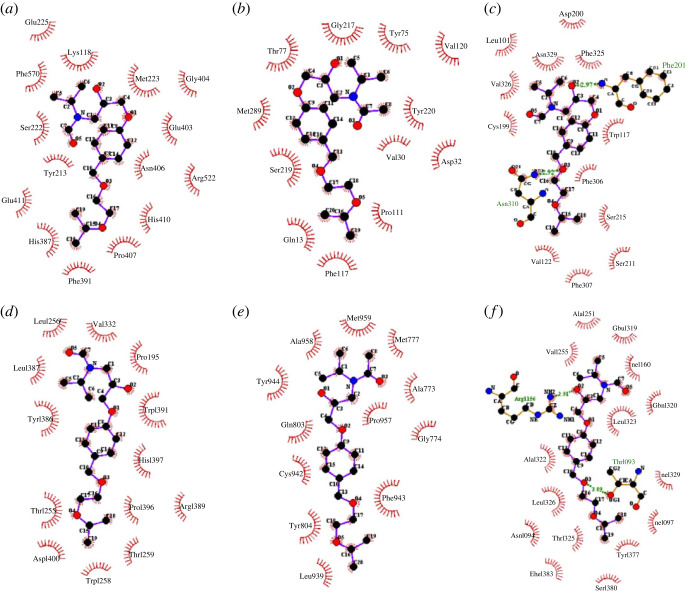
Visualization of complexes formed between: (*a*) *N*-formyl bisoprolol and 1O8A, (*b*) *N*-acetyl bisoprolol and 2V0Z, (*c*) *N*-formyl bisoprolol and 4BVN, (*d*) *N*-formyl bisoprolol and 6JP5, (*e*) *N*-acetyl bisoprolol and 6L88, and (*f*) *N*-formyl bisoprolol and 7BVQ.

The 4BVN protein–ligand interaction exhibited the highest binding energy value among the six target proteins. Figure 2 illustrates the two-dimensional visualization of the ligand–receptor complex for the ligand with the most optimal ability to inhibit each protein.

### 3.3. Molecular dynamics

**Figure 3.**
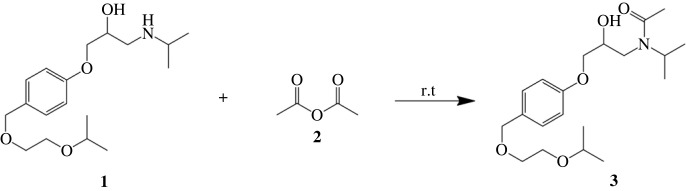
Reaction scheme for the synthesis of the *N*-acetyl bisoprolol compound.

**Figure 4.**
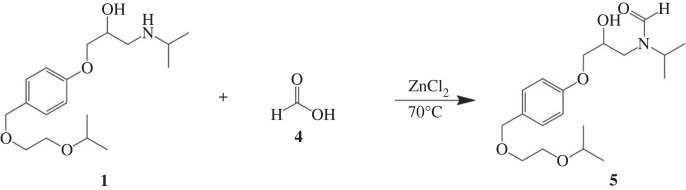
Reaction scheme for the synthesis of the *N*-formyl bisoprolol compound.

MD simulations lasting 100 ns were conducted to investigate the flexibility of the protein and the influence of the solvent (water) on the binding modes of compounds **1**, **3** and **5** with the 4BVN protein. It is a beta-adrenergic receptor which is responsible for increasing blood pressure when the amount of adrenaline hormone exceeds normal limits [25]. The ligand properties are shown in tables 8 and 9 for chemical properties and figures 3 and 4 for chemical structures, namely compounds **1**, **3** and **5**.

**Table 8.** Data for Lipinski's rule evaluation.

molecule	bisoprolol	*N*-acetyl bisoprolol	*N*-formyl bisoprolol
MW (g mol^−1^)	325.44	367.48	353.45
fraction Csp3	0.67	0.65	0.63
rotatable bonds	12	13	13
HBA	5	5	5
HBD	2	1	1
TPSA (Å^2^)	59.95	68.23	68.23
XLOGP3	1.87	1.87	1.66
ESOL Log S	−2.44	−2.61	−2.4

**Table 9.** Data for the ADMET evaluation.

molecule	bisoprolol	*N*-acetyl bisoprolol	*N*-formyl bisoprolol
ESOL solubility (mg ml^−1^)	1.19	9.04 × 10^−1^	1.42
GI absorption	high	high	high
BBB permeant	yes	yes	yes
Pgp substrate	yes	yes	no
CYP1A2 inhibitor	no	no	no
CYP2C19 inhibitor	no	no	no
CYP2C9 inhibitor	no	no	no
CYP2D6 inhibitor	yes	yes	yes
CYP3A4 inhibitor	no	no	no
log *Kp* (cm s^−1^)	−6.96	−7.21	−7.28

MD simulations were also conducted to validate the binding energy from molecular docking simulations. The results of binding energy from MD are shown in table 10.

**Table 10.** Binding energy scores from molecular dynamics simulations.

protein	binding energy (kJ mol^−1^)		
	bisoprolol	*N*-acetyl bisoprolol	*N*-formyl bisoprolol
1O8A	−131.781	−87.065	−116.498
2V0Z	−123.886	−29.012	−11.783
4BVN	−40.073	101.463	14.507
6JP5	−7.597	34.014	8.154
6L88	1.157	61.422	99.149
7BVQ	6.286	−2.97	−5.722

Based on simulations carried out by YASARA software, a more positive binding energy indicates a stronger interaction and bond between ligand and protein receptor. The binding energy values were obtained from MD simulations with six receptors that had been docked with ligands, namely compounds **1**, **3** and **5**. The results show that modified compounds **3** and **5** have more positive binding energy than commercial compound **1**, except for receptor 7BVQ. Of the six receptors used, two of them are beta-1 adrenergic receptors, namely 4BVN and 7BVQ. Modified compounds have better binding energy with 4BVN than 7BVQ. Therefore, further analysis regarding the MD simulation results was carried out for the complex with the 4BVN receptor.

**Figure 5.**
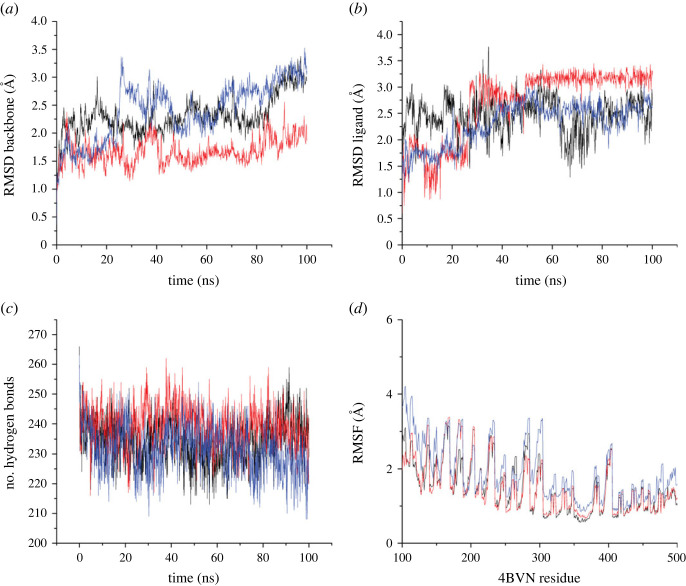
(*a*) RMSD of the 4BVN complex backbone, (*b*) RMSD of the 4BVN complex ligand, (*c*) number of hydrogen bonds in the 4BVN complex, and (*d*) RMSF of the 4BVN complex. Colour code: black for bisoprolol (compound **1**), red for *N*-acetyl bisoprolol (compound **3**) and blue for *N*-formyl bisoprolol (compound **5**).

The stability of the ligand–protein complexes was assessed by calculating the RMSD value during the simulation. Figure 5*a* illustrates that the backbone of the 4BVN protein in each complex experienced fluctuations within the range of approximately 1.5–3.8 Å. Both compounds **1** and **5** exhibited slight RMSD fluctuations and remained stable throughout the entire simulation period. By contrast, the RMSD of compound **3** showed a moderate increase at around 25 ns, peaking at approximately 3.25 Å, followed by a slight decrease, and subsequently maintained stability with minor fluctuations until 100 ns.

On the other hand, figure 5*b* demonstrates the RMSD ligand value of complex 4BVN with compounds **1, 3** and **5**. These complexes witnessed moderate fluctuations during the simulations from approximately 0 to 25 ns. Both compounds **3** and **5** remained steady with slight fluctuations between 45 ns and 100 ns with RMSD values at 3.0 and 2.5, respectively. On the other hand, complex 4BVN with compound **1** remained steady with moderate fluctuations during the simulation from 0 ns to approximately 60 ns and witnessed a moderate drop by approximately 74 ns with about 1.5 value of RMSD. Then, the RMSD value increased moderately to about 2.5 at 68 ns. From this point onwards, the value of RMSD remained steady with a moderate fluctuation until final at 100 ns.

The number of hydrogen bonds formed by compounds **1**, **3** and **5** in the 4BVN binding pocket was evaluated during the simulation period after approximately the first 2 ns of equilibration. The corresponding number of hydrogen bonds is presented in figure 5*c* and electronic supplementary material, table S1. Based on figure 5*c* and electronic supplementary material, table S1, the average number of hydrogen bonds in each complex during the simulation was 239, 234 and 231 for the **3**–4BVN > **1**–4BVN > **5**–4BVN complex compounds, respectively.

The hydrogen bond distance is a parameter that determines the strength of hydrogen bonds in the ligand–protein complex, with shorter distances indicating stronger hydrogen bonds. The hydrogen bond distances observed in the **5**–4BVN, **3**–4BVN and **1**–4BVN complex compounds were 1.868, 1.985 and 2.01 Å, respectively. Based on these results, it can be concluded that the **5**–4BVN complex compound exhibited the highest stability with the strongest hydrogen bonds throughout the simulation.

The root mean square fluctuation (RMSF) analysis was carried out to discover the flexibility of amino acid residues on the protein target during the simulation. It is often used to evaluate whether a structure is stable in the time scale of the simulations or is diverging from the initial coordinates [26]. Figure 5*d* illustrates the RMSF value of three compounds **1**, **3** and **5** with the 4BVN residue. Overall, it can be seen that the fluctuations of these compounds remained steady with small fluctuations. Compound **5** has the highest fluctuations. Moreover, compounds **1** and **3** have similar fluctuations during the simulations between residue 100 and 500. In terms of compound **5** with the highest fluctuations, it has the highest fluctuations at about residue 100 with a value of RMSF of about 4.2. In addition, its compound has the lowest fluctuations at about residue 210 and RMSF value of around 2.8. At that point, compounds **1** and **3** have a higher fluctuation than compound **5**. Regarding the other two compounds with the lower fluctuation, compound **3** has a lower fluctuation than compound **1** at residues roughly between 100 and 270 of 4BVN. From this point, compound **1** has a lower fluctuation than compound **3** at approximately 270–420 residue of 4BVN. After that, the lowest fluctuation is shown by compound **3**. Based on these results, it can be concluded that the **3**–4BVN complex has the smallest fluctuation during the simulation with 4BVN residue which means its compound exhibited the highest stability.

### 3.4. Lipinski's rule and ADMET prediction of bisoprolol derivatives

In addition to assessing the potential of a compound as a drug candidate based on its ability to inhibit disease-associated enzymes or proteins, its properties upon entering the body are also crucial considerations. SwissADME serves as a valuable tool for evaluating physicochemical descriptors and predicting ADMET parameters. ADMET encompasses the characteristics of absorption, distribution, metabolism, excretion and toxicity of a drug candidate compound. The results obtained from the SwissADME analysis are presented in tables 8 and 9.

There are five Lipinski rules which suggest that, in general, an orally active drug should not violate more than one of the following criteria: MW ≤ 500, number of H bond donors ≤ 5, number of H bond acceptors ≤ 10, mLogP ≤ 4.15 [18,27,28], rotatable bonds ≤ 10 and TPSA ≤ 140 [27,29]. Based on the data obtained, the two derivative compounds can be considered orally active drugs and compliant with Lipinski's rule.

Pgp substrate (P-glycoprotein) is an essential transporter that significantly impacts the pharmacokinetic properties of drug candidates. If a drug candidate can bind to Pgp substrate, it may interfere with its pharmacokinetics [30]. Pharmacokinetics describes how drugs are absorbed, distributed, metabolized and eliminated in the body [31]. Based on the results of the three compounds, only *N*-formyl bisoprolol did not exhibit the ability to inhibit Pgp substrate, while commercial bisoprolol and *N*-acetyl bisoprolol showed inhibition potential. This suggests that *N*-formyl bisoprolol is less likely to affect the absorption of other substances in the body.

Drug metabolizing enzymes play a crucial role in drug metabolism. The cytochrome P450 (CYP) family, particularly CYP450 3A4, is a significant detoxifying enzyme in the human body, especially in the liver [24]. From the results, both derivatives exhibit similar properties to commercial bisoprolol concerning the CYP family. However, while these three compounds can bind to CYP2C9 inhibitors, they do not affect the primary enzyme CYP3A4. This suggests that the three candidate drugs are unlikely to interfere with CYP metabolism.

## 4. Conclusion

This study aimed to develop bisoprolol derivatives as potential antihypertensive drugs using a combination of synthesis and *in silico* methods. The successful synthesis of *N*-acetyl bisoprolol and *N*-formyl bisoprolol was achieved through acetylation and formylation reactions of bisoprolol. These compounds exhibited docking scores similar to or higher than that of bisoprolol, the reference drug, indicating their potential as effective inhibitors. *In silico* methods, including molecular docking and MD simulations, were employed to assess the interaction of the synthesized compounds with various protein targets. The results showed that both *N*-acetyl bisoprolol and *N*-formyl bisoprolol formed stable complexes with the 4BVN protein, as evidenced by their high binding energies and consistent hydrogen bond distances during the MD simulations. Furthermore, the analysis of Lipinski's rule confirmed that these bisoprolol derivatives complied with the criteria for orally active drugs, further supporting their potential as drug candidates. Moreover, the ADMET prediction suggested that these compounds are less likely to interfere with Pgp substrate and CYP3A4 metabolism, indicating their favourable pharmacokinetic properties. Overall, this study provides valuable insights into the development of bisoprolol derivatives as promising antihypertensive drugs, paving the way for further experimental validation and potential future clinical applications.

## Data Availability

Supplementary material is available online [32].
